# Hemorrhagic fever with renal syndrome with secondary hemophagocytic lymphohistiocytosis in West China: a case report

**DOI:** 10.1186/s12879-019-4122-0

**Published:** 2019-06-04

**Authors:** Xiaoling Yang, Chuan Wang, Libo Wu, Xiaoqian Jiang, Sumei Zhang, Fuchun Jing

**Affiliations:** Department of Infectious Diseases, Baoji People’s Hospital Affiliated to Yan’an University, Baoji, 721000 Shaanxi province China

**Keywords:** Hemorrhagic fever with renal syndrome, *Hantaan virus*, Secondary hemophagocytic lymphohistiocytosis, Prognosis

## Abstract

**Background:**

Hemophagocytic lymphohistiocytosis (HLH) is a life-threatening disease characterized by an excessive systemic inflammatory response, which can be classified as primary HLH (pHLH) and secondary HLH (sHLH). Viruses are the primary pathogens causing sHLH. Hemorrhagic fever with renal syndrome (HFRS) is a rodent-borne disease caused by hantaviruses. Its main characteristics include fever, circulatory collapse with hypotension, hemorrhage, and acute kidney injury. The case of HFRS presented with sHLH is very rare in clinic. We reported the HFRS inducing by *Hantaan virus* (HTNV) presented with sHLH as the first case in Shaanxi province of west China.

**Case presentation:**

A case of HFRS in 69-year-old Chinese woman, which had persistent fever, cytopenia, coagulopathy, ferritin significantly increased, hepatosplenomegaly and superficial lymphadenopathy. The hemophagocytosis was found in bone marrow, which was consistent with the characteristics of the HLH. The patient recovered completely after timely comprehensive treatments.

**Conclusions:**

HTNV should be considered as one of the underlying viruses resulting in hemophagocytosis, and if occurs, the early diagnosis and rapid therapeutic intervention are very important to the prognosis of sHLH.

## Background

Hemophagocytic lymphohistiocytosis (HLH) is a reactive, hyperplastic disease of mononuclear phagocyte system, which is characterized by an excessive systemic inflammatory response. HLH can be classified as primary and secondary ones. The primary HLH (pHLH) is relatively rare and often occurs in infants aged 0–2 years, accompanied by genetic mutations and poor prognosis. The secondary HLH (sHLH), oppositely, is usually induced by a variety of medical conditions, including infections, autoimmune diseases, malignancies, immunodeficiencies and hematopoietic stem cell or organ transplantations, often associated with a better prognosis if recognized early [1]. Viruses, such as Epstein-Barr virus (EBV), adenovirus and herpes virus, are the primary pathogens causing sHLH. Occasionally, the bacterial, fungal, or parasitic infections may also cause it [2]. In most cases, sHLH patients could be treated successfully if the diagnosis and therapeutic intervention are timely [3].

Hemorrhagic fever with renal syndrome (HFRS) is a rodent-borne disease caused by hantaviruses, which is primarily characterized by fever, circulatory collapse with hypotension, hemorrhage and acute kidney injury [4]. HFRS mainly distributes in Asian and European countries [5]. In Europe, the most important serotype of hantaviruses is *Puumala virus* (PUUV) [6] but in Asia, *Hantaan virus* (HTNV) and *Seoul virus* (SEOV) are the common ones [7]. Though in China there is another serotype, *Dobrava-Belgrade virus* (DOBV), HTNV is still the commonest pathogen resulting in HFRS in Shaanxi province of west China [8].

The case of HFRS presented with sHLH is very rare in clinic. In the past decades, only two cases have been reported in France and South Korea respectively [9, 10]. There is no such a case reported in China, especially in west Shaanxi province. We provided the first case of HFRS with sHLH in this region.

## Case presentation

**Fig. 1:**
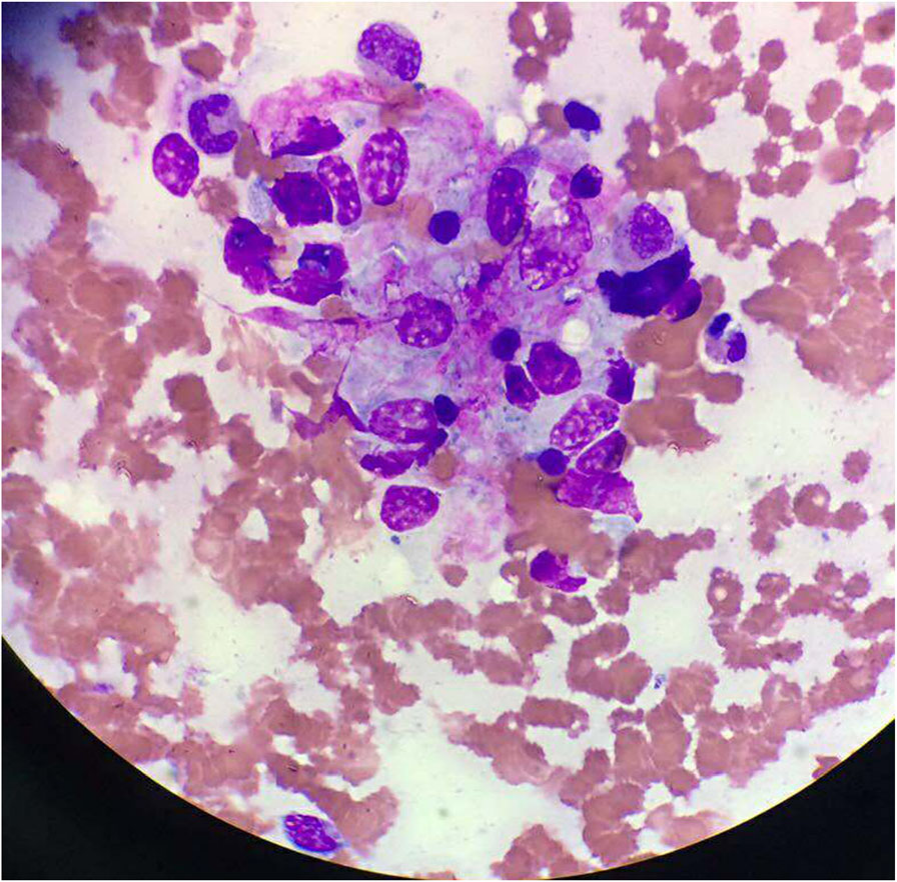
Bone marrow aspirate showing phagocytosis of neutrophil, nucleated erythrocyte, and platelets by benign histiocytes (Wright’s stain, × 100)

A 69-year-old Chinese woman was admitted to our hospital with fever and myalgia persisting for 2 days on August 12, 2017. The patient was not treated at a local clinic. Except a history of hypertension for 3 years, she had no other specific diseases or familial medical history. She lived in a rural area from her birth and often worked in fields. The vital signs were body temperature 39.0 °C, heart rate 122/min, respiratory rate 23/min and blood pressure 130/80 mmHg. The positive physical examination included a poor general condition, petechiae in the chest, palpable lymph nodes in the neck and axilla, and rough breathing sounds in lungs. Upon admission, hematologic tests revealed that her leukocyte count was 1.1 × 10^9^ /L, erythrocyte count 2.88 × 10^12^ /L, hemoglobin 102 g/L, platelet count 36.0 × 10^9^ /L, and abnormal lymphocytes 3%. Blood biochemistry showed blood urea nitrogen 14.17 mmol/L, creatinine 135.1 μmol/L, lactic dehydrogenase 989.4 IU/L, ferritin > 2000 μg/L and procalcitonin 66.29 ng/ml. The prothrombin time was 18.7 s, partial thromboplastin time 86.3 s, fibrinogen 1.90 g/L and D-Dimer over 20 μg/ml. Enzyme-linked immunosorbent assays of IgM and IgG antibodies for HFRS were both positive and the serotype of hantaviruses was HTNV. Additional serologic tests showed that antibodies against EBV, cytomegalovirus, herpes, adenovirus, respiratory syncytial virus, influenza virus A and B, human immunodeficiency virus, Hepatitis A, B, and C viruses, *Legionella pneumophila*, mycoplasma pneumoniae, chlamydia pneumoniae and rickettsia were negative. The scan of chest and abdomen by computed tomography demonstrated that hypostatic pneumonia and hepatosplenomegaly. The ultrasound examination to superficial lymph nodes revealed that multiple lymphadenectasis in the neck, axilla and groin. After 4-day hyperthermia (a peak temperature of up to 42 °C) from admission, the patient’s condition deteriorated gradually. Blood test showed that leukocyte count was 2.6 × 10^9^ /L, erythrocyte count 1.91 × 10^12^ /L, hemoglobin 70 g/L, platelet count 10.0 × 10^9^ /L, abnormal lymphocytes 18%, blood urea nitrogen 13.64 mmol/L, creatinine 200.6 μmol/L, lactic dehydrogenase 1169.0 IU/L, alanine aminotransferase 63.4 U/L, aspartate aminotransferase 260.7 U/L, albumin 28.3 g/L, creatine kinase 1859.4 U/L, creatine kinase-MB 58.3 IU/L, hydroxybutyrate dehydrogenase 816.0 IU/L, HDL-cholesterol 0.51 mmol/L, LDL-cholesterol 0.38 mmol/L, total cholesterol 1.34 mmol/L, triglyceride 1.04 mmol/L, the prothrombin time 18.5 s, partial thromboplastin time 100.2 s and fibrinogen 1.34 g/L. For clear comparison and understanding, the results of these blood parameters with their normal range on admission day 1 and day 4 were listed in Table 1. The immature cells and nucleated erythrocytes were found by peripheral blood smears. The concurrently cultures of blood, urine and sputum did not reveal any pathogen. The formation of histiocytes with prominent hemophagocytosis was discovered through bone marrow aspiration (Fig. 1).

**Table 1:** Laboratory tests of the patient on admission day 1 and day 4

Laboratory tests	Day 1	Day 4	Normal range
leukocyte count (× 10^9^ /L)	1.1	2.6	3.5–9.5
erythrocyte count (× 10^12^ /L)	2.88	1.91	3.8–5.1
hemoglobin(g/L)	102	70	115–150
platelet count (×10^9^ /L)	36	10	125–354
abnormal lymphocytes (%)	3	18	0–2
ALT (U/L)	–	63.4	7–45
AST (U/L)	202	260.7	13–40
LDH (U/L)	989.4	1169	109–245
BUN (mmol/L)	14.17	13.64	2.86–8.2
Creatinine (μmol/L)	135.1	200.6	40–90
PT (seconds)	18.7	18.5	10.5–14.5
APTT (seconds)	86.2	100.2	25–45
Fibrinogen (g/L)	1.9	1.34	2–4
Ferritin (μg/L)	>2000	–	15–650
Triglyceride (mmol/L)	–	1.04	0–2.3

*ALT* alanine aminotransferase, *AST* aspartate aminotransferase, *BUN* blood urea nitrogen, *LDH* lactic dehydrogenase, *PT* prothrombin time, *APTT* partial thromboplastin time

In the treatment of the patient, we just focused on the original disease HFRS, mainly taking the measurements of antiviral agent ribavirin, diuretic and intermittent hemodialysis in the initial and oliguric stages, antibacterial drug cefoperazone sodium and sulbactam sodium in dealing with pulmonary infection, maintenance of water and electrolyte acid-base balance and other supportive therapies when needed. No corticosteroids and specific therapy were applied. The patient recovered completely after the above comprehensive treatments on day 26. Bone marrow aspiration was performed again on September 7 and the result only showed secondary anemia but no signs of hemophagocytosis. The 3 months follow-up blood tests after her discharge from our hospital demonstrated normal outcomes.

## Discussion and conclusions

HFRS is mainly caused by four kinds of viruses, HTNV, PUUV, SEOV and DOBV, each of which may result in a severe outcome as its high mortality. The common clinical features of HFRS include fever, hypotension or shock, hemorrhage and acute kidney injury [4]. Though there were many researches probed the pathogenesis of this globally widespread disease in the past decades, there is still not a satisfied answer found to date [11]. Some believed that the infection of HTNV could induce human vascular endothelial injury and increase vascular permeability, which formed the pathological basis of HFRS and led to plasma exosmosis [12, 13]. Some thought that the viral infection could trigger the immune response such as T cell reaction, B cell reaction and cytokine storm, which may damage the vascular endothelium and induce plasmocyte into immature leucocyte [14–16]. This can partly explain the increase of the amount of immature leucocyte in the early stage of HFRS. Whatever the pathogenesis is, our report provided such a case with clear diagnosis of severe HFRS according to the detection of the antibody of HTNV and the clinical features and other laboratory or imaging tests.

HLH is a life-threatening disease characterized by an excessive systemic inflammatory response. Its prognosis is generally poor and the fatality rate could be as high as 50% [1]. The primary presentations of HLH in clinic are unexplained fever, hepatosplenomegaly, lymphadenopathy, cytopenia and coagulopathy. Hemophagocytosis in the bone marrow, spleen and lymph nodes is also an important diagnostic clue. In our case, the patient had persistent fever, hepatosplenomegaly, cytopenia, hypofibrinogenemia, increased blood levels of ferritin and hemophagocytosis in the bone marrow, which were completely consistent with the diagnostic criteria of HLH listed in Table 2 [17]. Besides, the patient had elevated blood lactate dehydrogenase, coagulopathy, abnormal liver function and superficial lymphadenopathy.

**Table 2:** Diagnostic Criteria of Hemophagocytic Lymphohistiocytosis (HLH) [17]

Molecular diagnosis of HLH or the presence of at least 5 of 8 criteria:	
1. Fever	
2. Splenomegaly	
3. Cytopenia (affecting at least 2 lineages in the peripheral blood), Hemoglobin levels < 90 g/L (in infants < 4 weeks old, hemoglobin < 100 g/L), Platelets < 100 × 10^9^ /L, Neutrophils < 1.0 × 10^9^ /L	
4. Hypertriglyceridemia and/or hypofibrinogenemia: Fasting triglycerides ≥3.0 mmol/L (ie, ≥ 265 mg/dl), Fibrinogen ≤1.5 g/L	
5. Documented hemophagocytosis in the bone marrow, spleen, or lymph nodes	
6. Low or absent natural killer cell activity	
7. Ferritin ≥500 μg/L	
8. Soluble CD25 (ie, soluble interleukin-2 receptor) ≥ 2400 U/ml	

The treatment of HLH is difficult, especially when it is secondary to another life-threatening disease such as HFRS. Timely diagnosis and treatment of the primary disease are vital to the prognosis of sHLH [18]. The use of antiviral agents, corticosteroids, immunoglobulin and even immune inhibitors such as etoposide and cyclosporine A is routine measurements in controlling the deterioration of the disease, especially in the treatment of EBV associated HLH [17]. Because the EBV related HLH is usually difficult to cure and fatal as its serious complications, e.g. hemorrhage, infection, or multiorgan failure [19]. However, in our case the patient was only cured with the administration of anti-infective agents, diuresis, hemodialysis and supportive therapies, no corticosteroids using in the treatment, indicating that the early diagnosis and comprehensive treatments to the primary infection is crucial to the prognosis of HLH secondary to HFRS. As sHLH is more often secondary to a variety of pathogen infections, including viruses, bacteria, fungi and parasites, rarely secondary to a same pathogen with a great amount, the further study of the pathogenesis of sHLH is usually very difficult in reality. So far, there were only two cases of sHLH after HFRS reported. Therefore, we can hardly know how the pathogenesis alters due to the activation of HLH by the HTNV.

In conclusion, we reported the first case of HFRS with sHLH caused by HTNV in China. That means HTNV should be considered as one of the underlying viruses resulting in hemophagocytosis, and if occurs, the early diagnosis and timely therapeutic intervention are very important to its prognosis.

## Data Availability

No applicable. All major data generated or analyzed during this study were included in this article.

## Abbreviations

DOBV: *Dobrava-Belgrade virus*
EBV: Epstein-Barr virus
HFRS: Hemorrhagic fever with renal syndrome
HLH: Hemophagocytic lymphohistiocytosis
HTNV: *Hantaan virus*
pHLH: primary Hemophagocytic lymphohistiocytosis
PUUV: *Puumala virus*
SEOV: *Seoul virus*
sHLH: secondary Hemophagocytic lymphohistiocytosis
